# Systematic review of three-dimensional printing for simulation training of interventional radiology trainees

**DOI:** 10.1186/s41205-021-00102-y

**Published:** 2021-04-21

**Authors:** Chase Tenewitz, Rebecca T. Le, Mauricio Hernandez, Saif Baig, Travis E. Meyer

**Affiliations:** 1 Mercer University School of Medicine, Savannah, GA USA; 2 University of Rochester School of Medicine and Dentistry, Rochester, New York USA; 3 UF Health Jacksonville, Jacksonville, FL USA

**Keywords:** Three-dimensional printing, Simulation training, High fidelity training, Interventional radiology

## Abstract

**Rationale and objectives:**

Three-dimensional (3D) printing has been utilized as a means of producing high-quality simulation models for trainees in procedure-intensive or surgical subspecialties. However, less is known about its role for trainee education within interventional radiology (IR). Thus, the purpose of this review was to assess the state of current literature regarding the use of 3D printed simulation models in IR procedural simulation experiences.

**Materials and methods:**

A literature query was conducted through April 2020 for articles discussing three-dimensional printing for simulations in PubMed, Embase, CINAHL, Web of Science, and the Cochrane library databases using key terms relating to 3D printing, radiology, simulation, training, and interventional radiology.

**Results:**

We identified a scarcity of published sources, 4 total articles, that appraised the use of three-dimensional printing for simulation training in IR. While trainee feedback is generally supportive of the use of three-dimensional printing within the field, current applications utilizing 3D printed models are heterogeneous, reflecting a lack of best practices standards in the realm of medical education.

**Conclusions:**

Presently available literature endorses the use of three-dimensional printing within interventional radiology as a teaching tool. Literature documenting the benefits of 3D printed models for IR simulation has the potential to expand within the field, as it offers a straightforward, sustainable, and reproducible means for hands-on training that ought to be standardized.

## Introduction

Simulation-based training is a method of experiential teaching that reproduces a real-world scenario in a controlled setting. Conventionally used to replicate high-risk tasks in areas such as military training, aviation, and aerospace professions [1], simulation-based training has become a prominent complement within medical education [2, 3]. Simulations can be performed outside of working hours, and certain procedural skills can be customized or repeated for a trainee’s needs, without compromising patient safety. Radiology residents and medical students are generally supportive of simulation use, as both groups have reported higher procedural confidence after completing a session on a number of diagnostic or interventional simulators [4–10].

Simulation training is especially relevant for procedural specialties like IR, where learners rely on haptic cues for skill acquisition [11]. There is an extensive amount of simulation options currently integrated into the medical field such as video games for inferior vena cava filter placement or percutaneous image-guided interventions [12, 13], phantom simulators for Computed Tomography (CT) biopsies [10, 14–16], and animal or cadaver models to practice endovascular access or interventions [17, 18]. Several studies have used simulators to demonstrate improved procedural technique, either in device manipulation [19–21], successful vessel cannulation [22], or reduced procedural time and radiation use [23–25]. There is burgeoning evidence demonstrating skill retention after the simulation [7], which has translated to improved patient outcomes. Such an example was documented by Andretta et al., who noted increased procedural proficiency and successful PICC line placement among interns who completed a simulation rotation on ultrasound-guided access [26].

Despite their advantages, simulations have several operational limitations and a correlative cost-benefit ratio. Simulation can be categorized by degree of situational realism, anatomic accuracy, or physiologic replication [27]. Although high-fidelity models have been shown to significantly increase technical performance in a simulated environment [28], they are often of higher cost, may have limited availability, or, if using animal or cadaver sources, can raise ethical concerns [18, 27, 29]. The reference standard in simulation medicine is the ability to develop a reproducible, realistic, and inexpensive product, which may be refined via three-dimensional (3D) printing. 3D printing has an emerging role within medicine and one of its primary benefits for the clinical realm includes the ability to produce patient-specific modeling from de-identified segmented stereolithography (STL) files, as this serves to augment several clinical applications, including trainee simulation [30, 31].

3D-printed models for simulation learning have been tested and positively received by trainees [32–36]. Some studies have found that 3D printed simulations increased student test scores when studying physiologic [34, 37] and pathologic [38–41] anatomy. 3D printed models have also facilitated groups of dental and surgical trainees in developing better preoperative plans [42, 43]. They have been shown to improve simulator procedural performance among anesthesia residents [44] and reduce fluoroscopy and simulator procedural times on endovascular aortic procedures with vascular surgery residents [45–47].

Radiology is well-poised to integrate 3D printing within simulation training. Prototypes, ranging from the lumbar spine to complex vasculature [48–51], have successfully been printed and can be modified for simulation purposes. While the role of three-dimensional printing for trainee education has been documented in other procedural specialties, there is a lack of knowledge about the role of 3D printing for trainee education within IR. Thus, the purpose of this review was to analyze the current articles that discuss the role of 3D printing for IR trainee education and emphasize the need for future experimentation.

## Methods

A literature query was conducted through April 2020 for articles discussing the use of 3D printing in IR simulation experiences via PubMed, Embase, CINAHL, Web of Science, and the Cochrane Library databases. Key search terms and boolean operators can be found in Table 1.

**Table 1:** Key search terms and boolean operators

Database	Search Terms	Number of articles
Cochrane	“3D printing” AND “simulation”	56
PubMed	“three-dimensional printing” AND (“radiology” OR “interventional radiology”) AND “simulation training”	214
	“three-dimensional printing” AND (“radiology” OR “interventional radiology”) AND “simulation training” AND “trainee”	40
EMBASE	“three dimensional printing” AND (“radiology” OR “interventional radiology”) AND “simulation training”	65
	“three dimensional printing” AND (“radiology” OR “interventional radiology”) AND “simulation training” AND (“trainees” OR “student”)	21
CINHAIL	“three dimensional printing” AND (“radiology” OR “interventional radiology”) AND (“simulation training” OR “simulation education” OR “simulation learning”)	83
	“three dimensional printing” AND (“radiology” OR “interventional radiology”) AND (“simulation training” OR “simulation education” OR “simulation learning”) AND (“trainees” OR “student”)	40
WEB OF SCIENCE	“three dimensional printing” AND (“radiology” OR “interventional radiology”) AND “simulation training”	207
	“three dimensional printing” AND (“radiology” OR “interventional radiology”) AND “simulation training” AND “trainees”	117

**Fig. 1:**
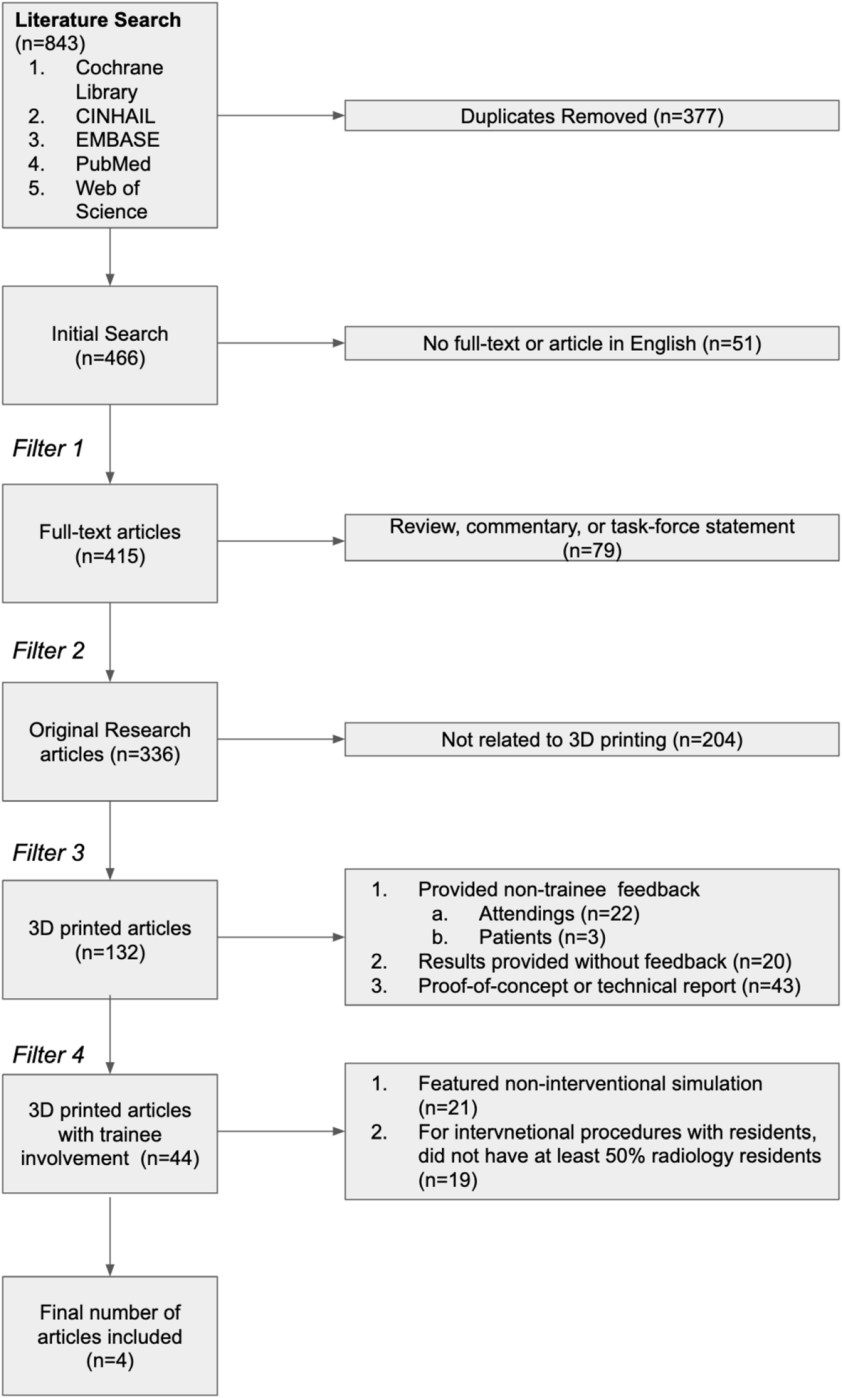
Systematic review flowchart

After removing duplicate records, non-English articles, and abstract-only articles, the remaining articles were reviewed. Articles were excluded if they did not feature the use of a 3D printed simulation models for interventional procedures by trainees, defined as either medical students, diagnostic radiology or interventional radiology residents, or interventional radiology fellows. For combined groups, more than 50% of the participants needed to identify as either medical students, diagnostic radiology or interventional radiology residents, or interventional radiology fellows. Figure 1 outlines the systematic review.

**Fig. 2:**
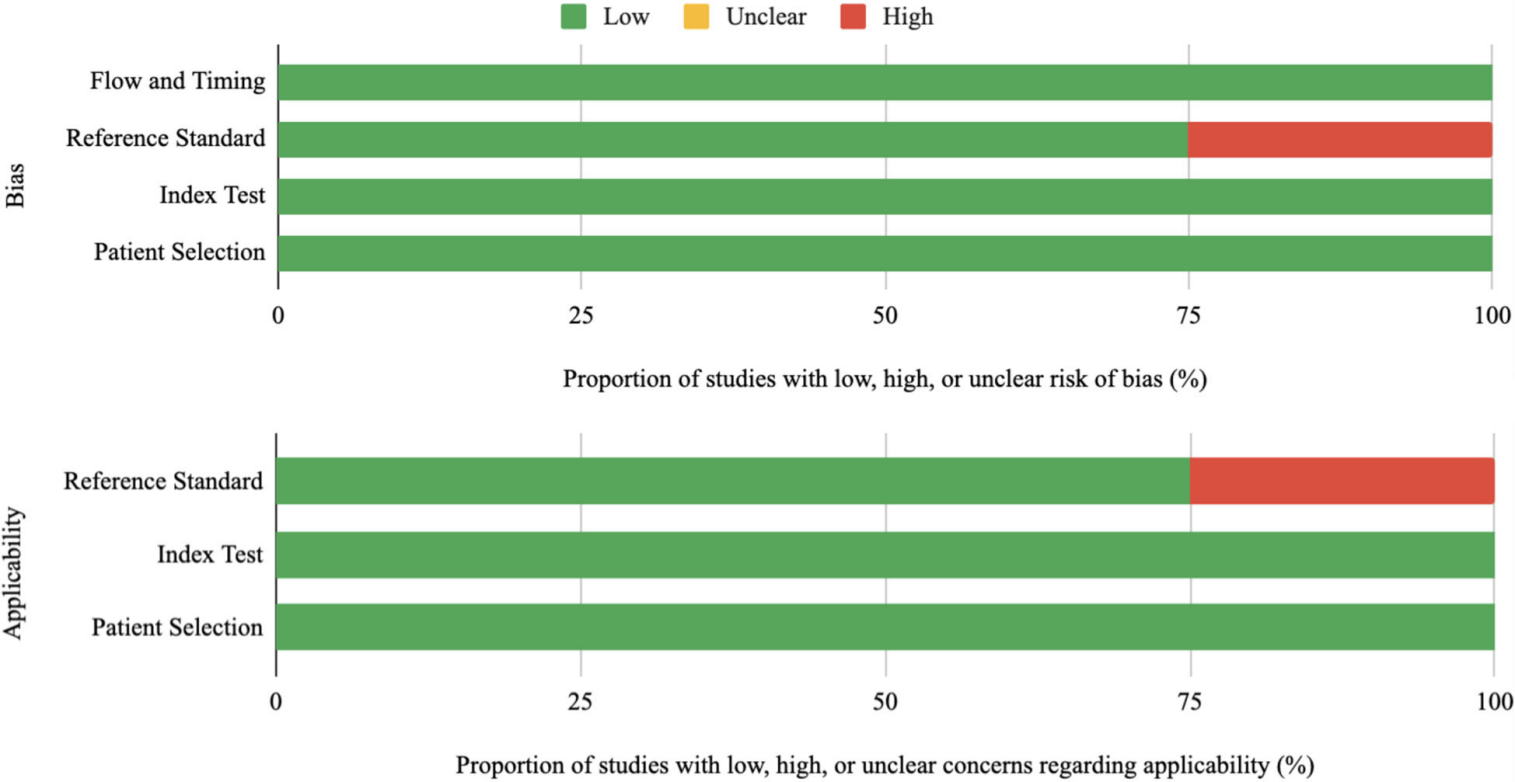
Summary of Quality Assessment of Diagnostic Accuracy Studies - 2 (QUADAS-2) ratings. The index test was the evaluation of the 3D-printed simulator. Applicability refers to appropriateness of the simulation to measure trainee ability. The reference standard was a comparison to evaluate the 3D-printed simulation. Flow and timing refers to equal treatment among participants and appropriate study follow-up, if applicable. There was a low to moderate degree of bias established due to the variability between study designs in the reviewed articles

To assess data quality, studies were evaluated by two reviewers using the Quality Assessment of Diagnostic Accuracy Studies - 2 (QUADAS-2) tool (Fig. 2) [52].. The index test was the evaluated 3D printed simulation in each study. The reference standard included a comparison to evaluate the 3D-printed simulation, which could include a pre- and post- assessment, control simulation, or outcomes from a control group. Two independent reviewers appraised these articles.

## Results

As noted in Fig. 1, the initial search yielded 843 records and was reduced to a total of 466 after removal of 377 duplicates. Of these, 51 of 466 records were further excluded due to a lack of full-text and availability of the record in English. The majority of excluded articles were original research articles that were not related to 3D printing. Of the 131 articles that discussed 3D printing, 44 of 141 (31.2%) of those articles featured trainee involvement, and 23 of the 44 (52.7%) of these articles featured a 3D printing procedure-oriented simulation. Among the remaining 23 articles, four articles featured our trainee population of interest (i.e. radiology or interventional radiology residents/fellows or medical students) and were included in the primary review.

The 19 remaining articles were excluded because they did not feature the target trainee population or the target trainee population was not represented by at least 50% of trainees in the study. The excluded articles implemented the use of 3D printed models for medical trainees in a variety of specialties. The predominant specialty that integrated 3D printing into its simulations was neurosurgery with seven articles [53–59]. These studies used 3D printed models of the cerebral vascular network to train neurosurgery residents and fellows on procedures such as cerebral aneurysm clipping and endoscopic ventriculostomy. Vascular surgery and anesthesiology were mentioned in three articles each [46, 47, 60–63]. The vascular surgery articles focused on endovascular repair of aneurysms and needle puncture of the gluteal artery using 3D printed vascular models while the anesthesiology articles used 3D printed models to simulate fiberoptic bronchoscopy procedures, thoracic spinal needle insertion, and central venous access [46, 47, 60–63]. Urology had a total of two articles where one of the articles featured interventional radiology trainees; however, this study did not meet the criteria of at least 50% IR trainee involvement [32, 64]. A 3D printed bladder was used to simulate urethrovesical anastomosis repair in one of the articles while the other article had a 3D printed human pelvicalyceal system, kidney, and adjacent structures to simulate percutaneous nephrolithotomy [32, 64]. The remaining articles had only one study associated with its specialty: cardiology (3D printed heart for electrophysiology training) [65], otolaryngology (3D printed model of the sinuses and skull base for anatomical education) [66], oral and maxillofacial surgery (3D printed model of the orbit for orbital surgery training) [67], and dentistry (3D printed model of pediatric dentition for pediatric dental education) [68].

All of the 19 excluded articles showed a general positive attitude toward 3D printed simulations where these models could be used for medical anatomy education as well as development of medical trainees’ procedural skills [32, 46, 47, 53–68]. The majority of these articles, 17 articles, tested the 3D simulations with trainees further in their medical career such as residents, fellows, or attendings [32, 46, 47, 53, 55, 57–68]. The remaining two articles consisted of medical students and residents [54, 56]. The higher level of trainees provides a more accurate assessment of particular values such as face, content and constructive validity due to the increased exposure to the real life procedures.

Out of the 19 omitted articles, two of the articles analyzed the face, content and construct validity of 3D printed simulators and revealed a positive trend in both studies [32, 58]. Weinstock et al. had a high face validity mean score at 4.69/5.00, a high content validity at 4.88/5.00, and a construct validity score that indicated that the 3D model provided a significant means to distinguish between novices and expert surgical skills when performing endoscopic ventriculostomy [58]. Ghazi et al. had similar results compared to Weinstock et al. with a high face validity at 4.5/5.0, a high content validity at 4.6/5.0, and a construct validity score that supports the theory that a 3D constructed percutaneous nephrolithotomy simulation provides a comprehensive tool for surgical skills development and evaluation before hand-on experience [32]. The high rated face, content, and construct validity ultimately emphasizes the benefits of 3D printed simulations and its practicality compared to real world procedures.

Although numerous articles reported a decreased cost of their 3D printed simulations [53, 54, 56], only two of the articles (anesthesia and cardiology) directly compared their 3D simulation to commercially available models [62, 65]. Pederson et al. noted a $110 3D printed bronchoscopic simulator compared to a commercial model priced greater than $2590 [62]. Seslar et al. featured a $2000 3D printed electrophysiology simulator compared to commercially available models valued at over $100,000 [65]. These two examples give a limited view of the possible cost benefits of 3D printing with medical training.

All of the criteria fulfilling articles reviewed were single institution studies with small sample sizes. The simulated procedures in the aforementioned articles included endoscopic biliary drainage [69], minimally-invasive CT-guided spine procedures [70], and ultrasound guided femoral artery access [71, 72]. Two of the studies analyzed the performance of IR procedures using medical students as the sole test population. Sheu et al. included a randomized assortment of medical students ranging from all years of training (*n* = 49) while Li et al. recruited first- and second-year medical students (*n* = 13). O’Reilly et al. invited a cohort of first year radiology physicians in training, (*n* = 19) for their study. Bundy et al. recruited the most diverse test population with varying backgrounds in medicine, including: technologists (*n* = 2), medical students (*n* = 1), residents (*n* = 2), fellows (*n* = 4) with consulting attending physicians (*n* = 2). To evaluate the effectiveness and provide a statistical method of comparison of simulation training using 3D printed materials, each study implemented a distinct approach. The heterogeneity in reported results was the main issue identified by QUADAS-2 assessment, which the reviewers assessed as a low to moderate degree of bias.

In addition to the similarities in the test populations, all four of the studies implemented a pre- and post-test questionnaire that utilized a Likert scale to quantify subjective opinions on 3D printing in IR training. Bundy et al. and Li et al. used a 10-point likert scale to assess the comfort with endoscopic techniques and lumbar punctures respectively. O’Reilly et al. used a 6-point Likert scale to assess the preference between a 3D printed and a commercially available femoral access model of the lower limb in teaching vascular access. Sheu et al. used a five-point likert scale to compare the ease of use, usefulness in practice and student confidence when using a 3D printed and commercially available phantom for femoral artery access.

All four of the studies reported positive feedback regarding the use of 3D printed models for trainee simulation. The comfortability and preference to use 3D printed models for endoscopic training increased in four different procedures as noted in Bundy et al.; however, the only statistically significant results included a 39.7% (*p* < 0.05) increase in comfortability performing endoscopic cholecystostomy and a 39.7% (p < 0.05) increase in the trainee’s likelihood to use endoscopy for percutaneous gastrostomy placement. Li et al. showed a significant increase in the confidence in needle stick placement for a control group, which was trained on a 3D model once, and a training group, which was trained on a 3D model twice. The confidence for the control group changed from 1.83 ± 2.0 to 5.8 ± 1.6 with *p* < 0.004 while the training group had a change in confidence from 1 ± 0 to 6.1 ± 1.1 with *p* < 0.00001. Additionally, there was a significant reduction in the number of needle stick placements for the training group when comparing the initial session, 8.0 ± 1.3 and the final session, 5.4 ± 1.5, with *p* < 0.005. The study further associated a 4.5 mGy-cm reduction of dose with a reduction of 3 needle readjustments, which correlates to a decreased reduction of radiation dose for the training group.

The preference to use the 3D printed model over a commercially available model for femoral artery access training was noted in O’Reilly et al. with an average rating of 5.1 out of 6. Sheu et al.’s results demonstrated that the majority of students in both the 3D printed ultrasound compatible vascular access model (3DPVAM) and commercial model (CM) groups agreed or strongly agreed that the models were useful for practice (96.2% and 95.7%, respectively; *p* < 0.87). Student confidence in performing femoral artery access increased by 2 Likert points in both trainee groups. Additionally, there was no difference in the average confidence change when comparing the training experience using the 3D printed model and the commercially produced model (*p* < 0.001).

The majority of the test populations in the excluded articles, 17 out of the 19 articles, consisted of higher-level medical professionals including residents and attendings [32, 46, 47, 53, 55, 57–68]. This contrasts with the 4 included articles where 2 out of the 4 studies used medical students as their test subjects [70, 72]. Although this provides a unique, unbiased perspective in the evaluation of the ability of the simulators to teach particular procedures, the medical students’ analysis may not accurately reflect the simulators’ realism and overall efficacy compared to the clinical procedures and its real-life application. Further research using residents, fellows and attendings would greatly benefit future studies on 3D printed simulations in IR. Future articles analyzing the benefits of 3D printed simulators would benefit from more stringent statistical analysis by including face, content, and construct validity. As noted in two of the articles, there was a positive trend in all three of the specified validity analyses [55, 65]. A common theme in the 4 included articles and the 19 excluded articles was the positive feedback in favor of the integration of 3D printed models in trainee simulations [32, 46, 47, 53–72]. The feedback was attained through post-simulator testing questionnaires. These evaluations had a diverse array of subjective questions ranging from how the subjects’ confidence changed in performing the procedure after training with the 3D model to the simulations’ ability to effectively mimic the real-life procedures.

## Discussion

A recent needs-assessment survey prioritized the development of ultrasound-guided or interventional procedures for simulation-based training [73]. Contrary to aforementioned evidence of existing IR simulators [10, 12–18], interventional-based simulations remain one of the least common training opportunities available [6]. A survey conducted by Matalon et al. found that 40% of radiology residents do not use simulations as part of their procedural training [6]. The limited number of available articles that features three-dimensional printing in IR simulation training highlights this area of unmet need within radiology training. While it is possible that we missed a relevant study for our review, this limitation was minimized by using free text and MeSH terms across multiple databases. The only articles we did not review were ones that either had no full-text article or were a non-English article, which comprised a minority (51/466 = 10.9%) of our excluded articles.

There is a lack of quantitative or validated simulation measures. Simulators may not always be able to decipher between experienced or novice operators [74, 75] and must be educationally validated to be useful. Mechanisms for validation include: face validity, content validity, concurrent validity, discriminant validity, and predictive validity, defined in Table 2 [76]. While each of our studies fulfilled face validity by demonstrating how the simulations appeared under imaging, only Li et al. evaluated content validity in evaluating the students’ abilities to complete the intervention (i.e. CT-guided facet block) [70]. Sheu et al. tested concurrent validity by comparing their 3D printed model to the FemoraLineMan CM (Simulab Corp, Seattle, Washington), a commercially available femoral access simulator [72]. This degree of variation in objective feedback raised concerns for a low to moderate degree of bias during QUADAS-2 evaluation.

**Table 2:** Measures of validity and their definitions for simulation-models

Type of Validity	Definition
Face validity	Measures the extent that the simulation represents its real-life model
Content validity	Measures the ability for the model to meet its training objectives
Concurrent validity	Compares the model to gold standards in ability to measure the same behaviors
Discriminant validity	Discerns the ability for a model to produce results different from another validated test (if it is intended to do so)
Predictive validity	Evaluates if the simulator accurately or positively correlates with performance in a non-simulated environment

A potential reason for a limited number of validation measures was that the study population was primarily medical students, as opposed to residents or fellows. Medical students’ lack of clinical experience compared to residents, fellows, or attendings minimizes confounders when evaluating procedural performance [17, 72]. While each study could circumvent its study population limitations by establishing face validity on appropriate imaging modalities, future studies evaluating 3D printed simulation will require more robust testing with residents and attendings, especially in regard to predictive validity. Current literature regarding predictive validity for preoperative planning is encouraging, as investigators have associated the use of 3D printed simulations or guides with reduced operative times [77, 78]. Within simulation literature, this appears to have only been evaluated in audiology trainees for proper hearing-aid placement [79], where trainees who practiced on a 3D printed simulator achieved a higher percentage of proper hearing-aid placement compared to those who practiced on the control model.

Given the strengths and limitations of the available literature, future studies focusing on 3D printed procedural simulation models for interventional radiology trainees could benefit from the inclusion of an adequate number of trainees particularly from senior years of training. Additionally, robust pre study baseline assessment using both subjective Likert scale questionnaires as well as objective measures of skill such as time to complete the procedure or number of attempts needed before success is crucial for post study result analysis. Ensuring the study can evaluate face, content and construct validity is valuable in generalizing the results to a larger population. Studies should have redundancy of raters if subjective observations are being made. Comparison of the 3D printed simulator with commercially available models including the cost analysis would be of great help to those trying to justify incorporating 3D printed simulation in their training program. Although many simulators do exist, the number of applications in interventional radiology remains limited. Table 3 outlines possible procedures that may benefit from 3D printed simulation training.

**Table 3:** Interventional Radiology procedures amenable to possible 3D printed simulation

Ultrasound guided vein identification, puncture and canalization	
- PICC line placement	
- IVC filter placement	
- Tunneled catheter line placement	
- Dialysis fistula and graft access	
Ultrasound guided arterial identification, puncture and canalization	
- Femoral artery access	
- Radial artery access	
Ultrasound guided biopsy and tissue sampling	
- Thyroid nodule fine needle aspiration	
- Renal lesion biopsy	
- Hepatic lesion biopsy	
CT guided biopsy and tissue sampling	
- Renal lesion biopsy	
- Hepatic lesion biopsy	
CT guided catheter placement	
- Abscess drain placement	
- Percutaneous cholecystostomy tube placement	
Fluoroscopic guided non-vascular procedures	
- Lumbar puncture	
- Joint arthrograms	
Fluoroscopy guided endovascular simulation	
- Coil or plug embolization placement and deployment	
- Stent placement and deployment	
- Endovascular aortic repair (EVAR) placement, gate canalization and deployment	

A common theme noted between these studies was the reduced production cost of these models compared to commercially available alternatives. The biliary simulation was one of the cheapest models to produce, costing approximately $170 for materials, while the lumbar spinal simulation, which required staged printing of bony structures, nerves, and vessels before assembly, was estimated to cost $5400. Sheu et al.’s femoral access model, with an estimated lifetime of approximately a dozen punctures, cost around $647 for the initial model and $100 to replace the 3D-printed vessels. This vastly differs from the competitive alternatives, which may cost between $2000–$4000, with replacement parts costing between $600 and $1600 [72]. This, however, does not factor the printer and software costs, which, again, widely varied between studies. Printed models first require segmentation, which can be completed on either free, open-source software or require purchasing high-end modeling software [30], while actual printers can range from $6000–$750,000 [80]. The financial cost and operational resources associated with 3D printing requires funding and institutional support, which may be another factor in the limited number of simulation articles for IR-related procedures. However, despite upfront investment, these costs can be shared across a hospital, and its resources, from preoperative modeling, simulations, and patient education, are applicable within multiple specialties [30, 31]. Although the costs of owning a printer for a particular lab can require a significant financial upfront investment, there are many various options to circumvent this issue. Many of the larger academic universities have their own 3D printing workshops, often housed in the central library and accessible to any member of campus. With this option in mind, a lab can send their designs into these workshops to help put together their particular devices. If the researchers’ organization does not have any in-house printers or specific materials, the product can be outsourced to a 3D printing service. Although owning a printer provides benefits such as on demand production and eventual lower costs, outsourcing provides its own unique benefits including no upfront investment, a wider array of 3D printing technologies and materials that can fit a certain criteria, and the availability of design expertise and recommendations when determining a design [81]. With these options in mind, it is also reasonable to consider using an in-house as well as an outsourced 3D printer based on the particular material needs as well as specific printer requirements of the project [81].

## Conclusion

Available literature, while supportive of the use of 3D-printing within vascular surgery, cardiology and for procedures performed by non-radiological specialties, is sorely lacking for interventional radiology. The paucity of literature regarding 3D-printing for simulation learning within IR reveals an essential area of improvement in medical education curricula. A lack of objective outcomes hinders advancements in simulation learning, to the disadvantage of trainees. Subsequent research and standardization of 3D-printed simulated experiences can offer more uniform and controlled learning opportunities for trainees to safely refine procedural techniques in the unique field of IR.

### Summary of points

- 3D printing has been utilized as a means of producing high-quality simulation models for trainees in procedure-intensive or surgical subspecialties.
- Less is known about its role for trainee education within interventional radiology.
- Our search identified a scarcity of published sources that appraised the use of three-dimensional printing for simulation training in IR where a total of four articles were found.
- Although presently available literature endorses the use of three-dimensional printing within interventional radiology as a teaching tool, there is a lack of studies that evaluates the predictive validity of 3D-printed simulation-based training in IR.
- Further research and integration of 3D-printed simulations into IR medical training can help develop and expand this field to a great extent.

## Data Availability

Not applicable.

## References

[1] Al-Elq AH (2010). Simulation-based medical teaching and learning. J Family Community Med.

[2] Kusunose K, Yamada H, Suzukawa R, Hirata Y, Yamao M, Ise T, Yagi S, Akaike M, Sata M (2016). Effects of transthoracic echocardiographic simulator training on performance and satisfaction in medical students. J Am Soc Echocardiogr.

[3] Mirza S, Athreya S (2018). Review of simulation training in interventional radiology. Acad Radiol.

[4] Alsafi A, Alsafi Z, Hamady MS (2015). TEVAR planning and simulation training improves trainee confidence. [P-189]. Cardiovasc Intervent Radiol.

[5] Hoang NS, Ge BH, Kuo WT (2020). Developing and evaluating a simulator for complex IVC filter retrieval. Acad Radiol.

[6] Matalon SA, Chikarmane SA, Yeh ED, Smith SE, Mayo-Smith WW, Giess CS (2019). Variability in the use of simulation for procedural training in radiology residency: opportunities for improvement. Curr Probl Diagn Radiol.

[7] Nitsche JF, Conrad S, Hoopes S, Carrel M, Bebeau K, Brost BC (2019). Continued validation of ultrasound guidance targeting tasks: assessment of internal structure. Acad Radiol.

[8] Orr KE, Hamilton SC, Clarke R, Adi MY, Gutteridge C, Suresh P, Freeman SJ (2019). The integration of transabdominal ultrasound simulators into an ultrasound curriculum. Ultrasound..

[9] Picard M, Nelson R, Roebel J, Collins H, Anderson MB (2016). Use of Low-Fidelity simulation laboratory training for teaching radiology residents CT-guided procedures. J Am Coll Radiol.

[10] Sekhar A, Sun MR, Siewert B (2014). A tissue phantom model for training residents in ultrasound-guided liver biopsy. Acad Radiol.

[11] Johnson SJ, Guediri SM, Kilkenny C, Clough PJ (2011). Development and validation of a virtual reality simulator: human factors input to interventional radiology training. Hum Factors.

[12] Jeun B, Ghassemi J, Pua BB (2012). Simulation training in inferior vena cava filter placement. [Abstract No. 440]. J Vasc Interv Radiol.

[13] Ghassemi J, Jeun B, Pua BB. Simulation training in percutaneous image-guided interventions. [Abstract No. 387]. J Vasc Interv Radiol. 23 (3, Suppl), S154 (2012).

[14] Baadh A, Fadl A, Georgiou N, Hoffmann JC. A pilot program for use of a homemade phantom for CT biopsy simulation training. [Abstract No. 376]. J Vasc Interv Radiol. 26 (2, Suppl), S167 (2015).

[15] Johnson SJ, Hunt CM, Woolnough HM, Crawshaw M, Kilkenny C, Gould DA, England A, Sinha A, Villard PF (2012). Virtual reality, ultrasound-guided liver biopsy simulator: development and performance discrimination. Br J Radiol.

[16] Villard PF, Vidal FP, ap Cenydd L, et al. Interventional radiology virtual simulator for liver biopsy. Int J Comput Assist Radiol Surg. 9 (2), 255–267 (2014). 10.1007/s11548-013-0929-0 23881251

[17] Meek MEM, Meek JC, Hollowoa B, Li R, Deloney LA, Phelan KD (2018). Lightly embalmed cadavers as a training tool for ultrasound-guided procedures commonly used in interventional radiology. Acad Radiol.

[18] McLeod H, Cox BF, Robertson J, Duncan R, Matthew S, Bhat R, Barclay A, Anwar J, Wilkinson T, Melzer A, Houston JG (2017). Human Thiel-embalmed cadaveric aortic model with perfusion for endovascular intervention training and medical device evaluation. Cardiovasc Intervent Radiol.

[19] Coates PJ, Zealley IA, Chakraverty S (2010). Endovascular simulator is of benefit in the Acquisition of Basic Skills by novice operators. J Vasc Interv Radiol.

[20] Powell DK, Jamison DK, Silberzweig JE (2015). An endovascular simulation exercise among radiology residents: comparison of simulation performance with and without practice. Clin Imaging.

[21] Saratzis A, Calderbank T, Sidloff D, Bown MJ, Davies RS (2017). Role of simulation in endovascular aneurysm repair (EVAR) training: a preliminary study. Euro J Vasc Endovasc Surg.

[22] Narra P, Kuban J, Grandpre LE, Singh J, Barrero J, Norbash A (2009). Videoscopic phantom-based angiographic simulation: effect of brief angiographic simulator practice on vessel cannulation times. J Vasc Interv Radiol.

[23] Dias TR, Alves Junior JDDC, Abdala N (2017). Learning curve of radiology residents during training in fluoroscopy-guided facet joint injections. Radiol Bras.

[24] Faulkner AR, Bourgeois AC, Bradley YC, Hudson KB, Heidel RE, Pasciak AS (2015). Simulation-based educational curriculum for fluoroscopically guided lumbar puncture improves operator confidence and reduces patient dose. Acad Radiol.

[25] Mendiratta-Lala M, Williams TR, Mendiratta V, Ahmed H, Bonnett JW (2015). Simulation center training as a means to improve resident performance in percutaneous noncontinuous CT-guided fluoroscopic procedures with dose reduction. AJR Am J Roentgenol.

[26] Andreatta P, Chen Y, Marsh M, Cho K (2011). Simulation-based training improves applied clinical placement of ultrasound-guided PICCs. Support Care Cancer.

[27] Amin A, Salsamendi J, Sullivan T (2019). High-Fidelity endovascular simulation. Tech Vasc Interv Radiol.

[28] Sidhu RS, Park J, Brydges R, MacRae HM, Dubrowski A (2007). Laboratory-based vascular anastomosis training: a randomized controlled trial evaluating the effects of bench model fidelity and level of training on skill acquisition. J Vasc Surg.

[29] Miller ZA, Amin A, Tu J, Echenique A, Winokur RS (2019). Simulation-based training for interventional radiology and opportunities for improving the educational paradigm. Tech Vasc Interv Radiol.

[30] Trace AP, Ortiz D, Deal A (2016). Radiology’s Emerging Role in 3-D Printing Applications in Health Care. J Am Coll Radiol.

[31] Ballard DH, Trace AP, Ali S, Hodgdon T, Zygmont ME, DeBenedectis CM, Smith SE, Richardson ML, Patel MJ, Decker SJ, Lenchik L (2018). Clinical applications of 3D printing: primer for radiologists. Acad Radiol.

[32] Ghazi A, Campbell T, Melnyk R, Feng C, Andrusco A, Stone J, Erturk E (2017). Validation of a full-immersion simulation platform for percutaneous Nephrolithotomy using three-dimensional printing technology. J Endourol.

[33] Goudie C, Kinnin J, Bartellas M, Gullipalli R, Dubrowski A (2019). The use of 3D printed vasculature for simulation-based medical education within interventional radiology. Cureus..

[34] Low CM, Morris JM, Matsumoto JS, Stokken JK, O’Brien EK, Choby G (2019). Use of 3D-printed and 2D-illustrated international frontal sinus anatomy classification anatomic models for resident education. Otolaryngol Head Neck Surg.

[35] Marconi S, Pugliese L, Botti M, Peri A, Cavazzi E, Latteri S, Auricchio F, Pietrabissa A (2017). Value of 3D printing for the comprehension of surgical anatomy. Surg Endosc.

[36] Ploch CC, Mansi CSSA, Jayamohan J, Kuhl E (2016). Using 3D printing to create personalized brain models for neurosurgical training and preoperative planning. World Neurosurg..

[37] Cai B, Rajendran K, Bay BH, Lee J, Yen CC (2019). The effects of a functional three-dimensional (3D) printed knee joint simulator in improving anatomical spatial knowledge. Anat Sci Educ.

[38] AlAli AB, Griffin MF, Calonge WM, Butler PE (2018). Evaluating the use of cleft lip and palate 3D-printed models as a teaching aid. J Surg Educ..

[39] Costello JP, Olivieri LJ, Su L, Krieger A, Alfares F, Thabit O, Marshall MB, Yoo SJ, Kim PC, Jonas RA, Nath DS (2015). Incorporating three-dimensional printing into a simulation-based congenital heart disease and critical care training curriculum for resident physicians. Congenit Heart Dis.

[40] Guitarte Vidauarre A, Karsenty C, Vincent R (2020). Teaching congenital cardiopathies through 3D printed models, is it always useful? [634]. Arch Cardiovasc Dis Suppl.

[41] Li Z, Li Z, Xu R, Li M, Li J, Liu Y, Sui D, Zhang W, Chen Z (2015). Three-dimensional printing models improve understanding of spinal fracture--a randomized controlled study in China. Sci Rep.

[42] Yao CJ, Chow J, Choi WWS, Mattheos N (2019). Measuring the impact of simulation practice on spatial representation ability of dentists by means of impacted mandibular third molar (IMTM) surgery on 3D printed models. Eur J Dent Educ.

[43] Zheng B, Wang X, Zheng Y, Feng J (2019). 3D-printed model improves clinical assessment of surgeons on anatomy. J Robot Surg.

[44] Bortman J, Baribeau Y, Jegenathan J (2018). Improving clinical proficiency using a 3-dimensionally printed and patient-specific thoracic spine model as a hepatic task trainer. Reg Anesth Pain Med.

[45] Barber SR, Kozin ED, Dedmon M, Lin BM, Lee K, Sinha S, Black N, Remenschneider AK, Lee DJ (2016). 3D-printed pediatric endoscopic ear surgery simulator for surgical training. Int J Pediatr Otorhinolaryngol.

[46] Kärkkäinen JM, Sandri G, Tenorio ER, Alexander A, Bjellum K, Matsumoto J, Morris J, Mendes BC, DeMartino RR, Oderich GS (2019). Simulation of endovascular aortic repair using 3D printed abdominal aortic aneurysm model and fluid pump. Cardiovasc Intervent Radiol.

[47] Torres IO, De Luccia N (2017). A simulator for training in endovascular aneurysm repair: the use of three dimensional printers. Eur J Vasc Endovasc Surg.

[48] Javan R, Bansal M, Tangestanipoor A (2016). A prototype hybrid gypsum-based 3-dimensional printed training model for computed tomography-guided spinal pain management. J Comput Assist Tomogr.

[49] Javan R, Ellenbogen AL, Greek N, Haji-Momenian S (2019). A prototype assembled 3D-printed phantom of the glenohumeral joint for fluoroscopic-guided shoulder arthrography. Skelet Radiol.

[50] Rynio P, Falkowski A, Witowski J, Kazimierczak A, Wójcik Ł, Gutowski P (2016). Simulation and training of needle puncture procedure with a patient-specific 3D printed gluteal artery model. J Clin Med.

[51] Silvestro E, Shellikeri S, Trahan S, Sze R, Cahill A (2020). Fabrication of a custom pediatric phantom for pediatric interventional radiology endovascular simulation and training: technical aspects. [Abstract No. 560]. J Vasc Interv Radiol.

[52] Whiting PF, Rutjes AWS, Westwood ME, Mallett S, Deeks JJ, Reitsma JB, Leeflang MM, Sterne JA, Bossuyt PM, QUADAS-2 Group (2011). QUADAS-2: a revised tool for the quality assessment of diagnostic accuracy studies. Ann Intern Med.

[53] Karakas AB, Govsa F, Ozer MA, Eraslan C (2019). 3D brain imaging in vascular segmentation of cerebral venous sinuses. J Digit Imaging.

[54] Liu Y, Gao Q, Du S (2017). Fabrication of cerebral aneurysm simulator with a desktop 3D printer. Sci Rep.

[55] Mashiko T, Kaneko N, Konno T, Otani K, Nagayama R, Watanabe E (2017). Training in cerebral aneurysm clipping using self-made 3-dimensional models. J Surg Educ.

[56] Ryan JR, Chen T, Nakaji P, Frakes DH, Gonzalez LF (2015). Ventriculostomy simulation using patient-specific ventricular anatomy, 3D printing, and hydrogel casting. World Neurosurg.

[57] Wang JL, Yuan ZG, Qian GL, Bao WQ, Jin GL (2018). 3D printing of intracranial aneurysm based on intracranial digital subtraction angiography and its clinical application. Medicine (Baltimore).

[58] Weinstock P, Rehder R, Prabhu SP, Forbes PW, Roussin CJ, Cohen AR (2017). Creation of a novel simulator for minimally invasive neurosurgery: fusion of 3D printing and special effects. J Neurosurg Pediatr.

[59] Zheng JP, Li CZ, Chen GQ, Song GD, Zhang YZ (2018). Three-dimensional printed Skull Base simulation for Transnasal endoscopic surgical training. World Neurosurg..

[60] Rynio P, Falkowski A, Witowski J, Kazimierczak A, Wójcik Ł, Gutowski P (2020). Simulation and training of needle puncture procedure with a patient-specific 3D printed gluteal artery model. J Clin Med.

[61] Bortman J, Baribeau Y, Jeganathan J, Amador Y, Mahmood F, Shnider M, Ahmed M, Hess P, Matyal R (2018). Improving clinical proficiency using a 3-dimensionally printed and patient-specific thoracic spine model as a haptic task trainer. Reg Anesth Pain Med.

[62] Pedersen TH, Gysin J, Wegmann A, Osswald M, Ott SR, Theiler L, Greif R (2017). A randomised, controlled trial evaluating a low cost, 3D-printed bronchoscopy simulator. Anaesthesia..

[63] Sappenfield JW, Smith WB, Cooper LA, Lizdas D, Gonsalves DB, Gravenstein N, Lampotang S, Robinson AR (2018). Visualization improves supraclavicular access to the Subclavian vein in a mixed reality simulator. Anesth Analg.

[64] Wong NC, Hoogenes J, Alharbi B, Vij S (2016). Robotic surgical skill acquisition in trainees: a randomized comparison of robotic simulation training and the transfer of skills to a simulated robotic surgical task in the operating room using a 3D printed model. J Urol.

[65] Seslar SP, Patton KK (2018). Initial experience with a novel electrophysiology mapping simulator. Pacing Clin Electrophysiol.

[66] Hsieh TY, Cervenka B, Dedhia R, Strong EB, Steele T (2018). Assessment of a patient-specific, 3-dimensionally printed endoscopic sinus and Skull Base surgical model. JAMA Otolaryngol Head Neck Surg.

[67] Lichtenstein JT, Zeller AN, Lemound J, Lichtenstein TE, Rana M, Gellrich NC, Wagner ME (2017). 3D-printed simulation device for orbital surgery. J Surg Educ..

[68] Marty M, Broutin A, Vergnes JN, Vaysse F (2019). Comparison of student's perceptions between 3D printed models versus series models in paediatric dentistry hands-on session. Eur J Dent Educ.

[69] Bundy JJ, Weadock WJ, Chick JFB, Srinivasa RN, Patel N, Johnson E, Khayat M, Jeffers B, Gemmete JJ, Srinivasa RN (2019). Three-dimensional printing facilitates creation of a biliary endoscopy phantom for interventional radiology-operated endoscopy training. Curr Probl Diagn Radiol.

[70] Li Y, Li Z, Ammanuel S, Gillian D, Shah V (2018). Efficacy of using a 3D printed lumbosacral spine phantom in improving trainee proficiency and confidence in CT-guided spine procedures. 3D Print Med.

[71] O'Reilly MK, Reese S, Herlihy T, Geoghegan T, Cantwell CP, Feeney RNM, Jones JFX (2016). Fabrication and assessment of 3D printed anatomical models of the lower limb for anatomical teaching and femoral vessel access training in medicine. Anat Sci Educ.

[72] Sheu AY, Laidlaw GL, Fell JC, Triana BP, Goettl CS, Shah RP (2019). Custom 3-dimensional printed ultrasound-compatible vascular access models: training medical students for vascular access. J Vasc Interv Radiol.

[73] Nayahangan LJ, Nielsen KR, Albrecht-Beste E, Bachmann Nielsen M, Paltved C, Lindorff-Larsen KG, Nielsen BU, Konge L (2018). Determining procedures for simulation-based training in radiology: a nationwide needs assessment. Eur Radiol.

[74] Berry M, Lystig T, Reznick R, Lönn L (2006). Assessment of a virtual interventional simulator trainer. J Endovasc Ther.

[75] Berry M, Reznick R, Lystig T, Lönn L (2008). The use of virtual reality for training in carotid artery stenting: a construct validation study. Acad Radiol.

[76] Dawson S (2006). Procedural simulation: a primer. J Vasc Interv Radiol.

[77] Ballard DH, Mills P, Duszak R, Weisman JA, Rybicki FJ, Woodard PK (2019). Medical 3D printing cost-Savings in Orthopedic and Maxillofacial Surgery: cost analysis of operating room time save with 3D printed anatomic models and surgical guides. Acad Radiol.

[78] Golab A, Smektala T, Kaczmarek K, Stamirowski R, Hrab M, Slojewski M (2017). Laparoscopic partial nephrectomy supported by training involving personalized silicone replica poured in three-dimensional printed casting Mold. J Laparoendosc Adv Surg Tech A.

[79] Koch RW, Saleh H, Folkeard P, Moodie S, Janeteas C, Agrawal SK, Ladak HM, Scollie S (2020). Skill transference of a probe-tube placement training simulator. J Am Acad Audiol.

[80] Computer Aided Technology. 3D Printer Price (2020). https://www.cati.com/3d-printing/3d-printer-price/

[81] Additive Manufacturing Execution System & Workflow Automation Software. 3D Printing In-House vs Outsourcing: the Definitive Guide (2018). https://amfg.ai/2018/07/04/3d-printing-outsourcing-vs-in-house/

